# Computational Aspects of Protein Functionality

**DOI:** 10.1002/cfg.360

**Published:** 2004-02

**Authors:** R. C. Paton, C.-H. Toh

**Affiliations:** 1 Department of Computer Science University of Liverpool Liverpool L69 3BX UK; 2 Department of Haematology University of Liverpool UK

## Abstract

The purpose of this short article is to examine certain aspects of protein functionality with relation to some key organizing ideas. This is important from a computational viewpoint in order to take account of modelling both biological systems and knowledge of these systems. We look at some of the lexical dimensions of the function and how certain constructs can be related to underlying ideas. The pervasive computational metaphor is then discussed in relation to protein multifunctionality, and the specific case of von Willebrand factor as a ‘smart’ multifunctional protein is briefly considered. Some diagrammatic techniques are then introduced to better articulate protein function.


**Comparative and Functional Genomics**
*Comp Funct Genom* 2004; **5**: 85–90.
Published online in Wiley InterScience (www.interscience.wiley.com). **DOI:** 10.1002/cfg.360

**Conference Review**

### Computational aspects of protein functionality

R. C. Paton[1]* and C.-H. Toh[2]
[1] *Department of Computer Science, University of Liverpool, UK*
[2] *Department of Haematology, University of Liverpool, UK*

**Correspondence to:*
*R. C. Paton, Department of Computer Science, University of Liverpool, Liverpool L69 3BX, UK.*
*E-mail: r.c.paton@csc.liv.ac.uk*

Received: 22 October 2003
Revised: 18 November 2003
Accepted: 20 November 2003

### Abstract

**The purpose of this short article is to examine certain aspects of protein functionality with relation to some key organizing ideas. This is important from a computational viewpoint in order to take account of modelling both biological systems and knowledge of these systems. We look at some of the lexical dimensions of the function and how certain constructs can be related to underlying ideas. The pervasive computational metaphor is then discussed in relation to protein multifunctionality, and the specific case of von Willebrand factor as a 'smart' multifunctional protein is briefly considered. Some diagrammatic techniques are then introduced to better articulate protein function. Copyright © 2004 John Wiley & Sons, Ltd.**

### Introduction

In order to describe, model and reason about biological function, with or without the use of computers, we need to be able to examine the nature and especially the use of the function concept. This short article examines a number of related tools of thought that can help with this examination.

### Describing biological functionality is a challenging problem

The notion of function can be overloaded with meanings and, as with many biological concepts, it is multidimensional in nature. Some of these dimensions are shown in Table 1, which summarizes a number of functional constructs in terms of their associated verbs. Some of these constructs, which can also be related to a number of underpinning systemic metaphors [12], deal with the relationship between the part and the whole, whilst others deal with the relationship of whole to part.

Computational challenges that have to be addressed when considering the issue of biological functionality include data- and knowledge-based system development and biosystems modelling. These various challenges are lexically and conceptually highly interrelated. Karp [8] reviewed some issues related to biological function and how it might be expressed or represented in computer information systems (e.g. in ontologies). His suggestion is to distinguish between local function and integrated function (i.e. parts in relation to wholes). The Gene Ontology Consortium [6] ontology distinguishes between, on the one hand, molecular function as what a gene product does and tasks that are performed, and on the other, biological process as the broad biological goals that are accomplished by ordered assemblies of molecular functions. So we might hope that local function and molecular function may be distinguished from integrated function and biological process. However, this may not be so easy to do in practice. It presumes that it is always possible to make a clear demarcation in organizational levels.

Functional and relational thinking allows us to talk and reason about the association of processes, causal relations and patterns of activity. This kind of thinking operates at a number of levels of scale in both time and space. Numerous authors have examined the status of function and its relations to goal, purpose, teleology and causality. Teleological language is concerned with concepts that access a

Copyright © 2004 John Wiley & Sons, Ltd.

**Table 1.** Functional constructs and associated verbs

| Functional construct | Some associated verbs |
|---|---|
| Niche | Occupy, fulfil, interact, work, provide, exchange ... |
| Role | Fulfil, (en)act, play, work, fulfil, perform, serve, operate, satisfy ... |
| Theme | Follow, flow, compose, direct, thread, act ... |
| Job | Work, do, achieve, perform, serve, operate, provide, task, pay ... |
| Welfare | Help, need, provide, enable, assist, facilitate, serve, require, satisfy ... |
| Affordance | Perceive, interpret, read, operate, measure, detect, influence ... |
| Factor | Effect, influence, change, exchange, contribute ... |
| Goal | Reach, find, attain, achieve, satisfy, target, maintain, direct ... |

number of pervasive ideas, such as entropy, least action, maxima, optima and search. Granit [7] interlinked closure and teleology when he argued that biological integration is interaction for a purpose. Teleonomic ideas transfer cybernetic and systems thinking to biology (e.g. [10]). Function statements presuppose a tendency to logic with regard to systems of closure and cyclicity, e.g. homeostasis defines closure properties. The relation between functional thinking and a logic of procedure was espoused by Cannon, who advocated an approach of establishing requirements and then proceeding to mechanisms.

Some have argued that biology should avoid functional language and thinking. A trivial approach is to recast functional language into a kind of function-free language. However, this may change the language but not necessarily the thinking processes. An alternative is to be aware of the underlying (underpinning) ideas of a domain of knowledge (e.g. the pervading ideas of teleology in physics). One pervading idea in contemporary biology is the computational metaphor.

### Protein functionality from an information-processing perspective

The concept of functionality is multidimensional. In this section we address one aspect of this functionality in terms of information processing. This analysis is orthogonal to the list of functional constructs in Table 1. In their book on aspects of the history of the protein sciences, Tanford and Reynolds [15] reflect on why they called their work *Nature's Robots*:

> ... robots are automatons — you don't need to tell them what to do, they already know. Proteins satisfy that criterion. For every imaginable task in a living organism, for every little step in every imaginable task, there is a protein designed to carry it out. And it is programmed to know when to turn on or off ... The common feature is that proteins are in control and know what to do without being told by the conscious mind ( [15] p. 3).

Automaton models of biochemical systems have been developed since the 1960s, e.g. Rosen's two-factor models of neurons and biochemical automata [13] and the algebraic models of Krohn, Langer and Rhodes [9]. More recently, biologists have noted similarities between cellular systems and adaptive computational networks (e.g. [1]). The scope for displacing information-processing descriptions into protein models is very broad, e.g. many proteins, such as enzymes and transcription factors, display 'cognitive' capacities, including memory capacity, pattern recognition, handling fuzzy data, multifunctionality, signal amplification, integration and crosstalk, and context-sensitivity (see e.g. [5]).

Conrad [3] discussed the idea of a seed-germination model of enzyme action which sought to take account of the multiplicities of interaction that give rise to enzyme function. This is a distinctly ecological notion in the sense that we are modelling the autecology of a molecular species. We may think of enzymes as 'smart thermodynamic machines' fulfilling a 'gluing' (functorial) role in the information economy of the cell. In that they are able to interact with other molecules in subtle and varied ways, we may say that many proteins display social abilities. The social dimension to enzyme agency also presupposes that proteins have an underlying ecology in that they interact with other molecules, including substrates, products, regulators, cytoskeleton, membranes, water as well as local electric fields (e.g. [17,16]). Using the metaphor of the cell-as-text (e.g. [11]), a variety of proteins, including enzymes and transcription factors, can be considered to be playing roles like the verbs of natural languages. They can be said to have cases that in natural language could be 'agent', 'location', 'source', 'destination' and so forth. Protein cases would include 'substrate', 'product', 'regulator(s)', 'locations', 'associations', 'co-agent(s)' and 'target site(s)'. Verb meanings

Copyright © 2004 John Wiley & Sons, Ltd. 

can be altered by prepositions and in an analogous sense proteins can be altered post-translationally by a range of mechanisms, including phosphorylation, methylation, glycosylation, myristoylation and so forth. The mood- and voice-like properties they exhibit can be related to their context-sensitivity and also to their internal configuration and localized interactions.

Given this brief review comment regarding information-processing aspects of proteins, we now consider one example of a 'smart' multifunctional protein that is involved in the information-rich process of regulating haemostasis; a fundamental biological and evolutionary mechanism pertinent to the prevention of death through haemorrhage.

### A smart multifunctional protein

Von Willebrand Factor (vWF) is a large molecular weight blood glycoprotein recognized as a multifunctional protein with regard to haemostasis or the process of arresting bleeding in response to injury [14]. With regard to its functionality, we may say that it mediates the adhesion of platelets to sites of vascular damage through binding to specific membrane glycoproteins of platelets and to constituents of exposed connective tissue (e.g. collagen). In addition, it 'senses' shear stresses in the fluid domain and 'adjusts' its conformation to reveal binding epitopes for requisite interactions with its multiple ligands. It also carries and stabilizes Factor VIII. There are also a number of possible functions for the propeptide of vWF, especially with regard to linking the process of inflammation to clot formation or coagulation.

Von Willebrand Factor is built from the assembly of subunits of 250 kDa each with 18% of molecular mass as carbohydrate. Each subunit comprises 2050 amino acids. Subunits are linked together into multimers that are over 20 000 kDa. The largest vWF multimeric forms are present in the subendothelial matrix and in platelet storage granules. In circumstances of injury, these forms are particularly functional for the binding of platelets and collagen when released from their storage sites into the immediate vicinity. The circulating form or plasma vWF is of a smaller average multimer size by comparison. Plasma factors that regulate the size of vWF multimers include a vWF cleaving protease (vWFCP) and thrombospondin-1 (TSP-1). These are relevant to the termination of vWF procoagulant activity at the point when bleeding has arrested.

Deficiency of vWF is the most commonly inherited bleeding disorder. This can be both quantitative and qualitative in nature. The importance of the requisite multimeric configuration is demonstrated when mutations interfering with multimerization generate haemostatic molecules that are ineffective. Conversely, changes leading to a gain in vWF function as a result of vWF-cleaving protease deficiency can lead to the formation of unusually large vWF multimers that can pathologically cause platelet-rich microthrombi. Clinically, this can manifest as thrombotic thrombocytopaenic purpura.

As such, the multifunctionality of vWF is modulated according to need and circumstance, disruption of which can have pathophysiological consequence. Some of the key functional capacities of vWF may be summarized as:

- SENSITIVE
  It is sensitive, not only to other molecular ligands but also to the fluid stresses within a vessel. The 'sensitivity' of this molecule affords it an information processing capacity that also enables us to think in terms of social behaviour.
- SOCIABLE
  vWF is a sociable molecule that is able to associate and aggregate with itself and also other molecules (e.g. collagen, heparin, and some platelet membrane glycoproteins). This adhering capability makes it like a 'glue' at a number of levels of description.
- BRIDGE
  This facilitates a bridging role at a semi-local level allowing the net(mesh)works of protein fibres and platelets to come together.
- CARRY and STABILIZE
  vWF acts as a carrier and stabilizer for factor VIII.
- GLUE
  At a local level, vWF acts as an adhesive by binding to other protein domains in collagen, heparin and surface molecules on the platelets. However, vWF is also part of a global glue when the process of coagulation activation becomes disseminated as a secondary response to septicaemia and trauma. Whilst this can be adaptive and protective to the host in the initial stages, maladaptation can sometimes occur

Copyright © 2004 John Wiley & Sons, Ltd. 

when regulatory mechanisms are overridden. In those circumstances, the adhesive property may lead to ischaemia due to vWF platelet-rich thrombi interrupting the circulation and causing end-organ damage.

So far, we have looked at ways in which language can enrich our concepts of protein multifunctionality (space prevents a more detailed discussion of multifunctional kinases, transcription factors, moonlighting proteins, 'underground' metabolism, and many other cases).

### Diagrammatic representations of vWF functionality

The language used to describe vWF functionality was applied to the molecule as a whole (or some abstract notion of the vWF species of molecule) and to some degree to specific parts of the molecule (such as binding). We now examine vWF functionality by looking at some of its functional domains (Figure 1a) through a number of diagrammatic representations.

The arcs of the non-directed 'star' graph on the left of Figure 1b are labelled with regard to four distinguishable aspects of vWF functionality. Further functional aspects could have been selected but we have chosen just four to make the process more understandable to a reader. The nodes can be thought of as representing the four component features of the molecule (e.g. represented by the domain description types) that afford these functions. The 'tetrahedron' graph on the right of the figure has been formed by taking each arc in the 'star' (source) graph and making it a node in the 'tetrahedron' (target) graph. The arcs in the target graph are relations and/or processes that are shared (or combine) source graph processes.

The graph to the left of the tetrahedron graph in Figure 2 has been generated by taking its arcs and making them nodes in the target graph (called a line graph). In this graph we see how possible patterns of interaction between different processes in

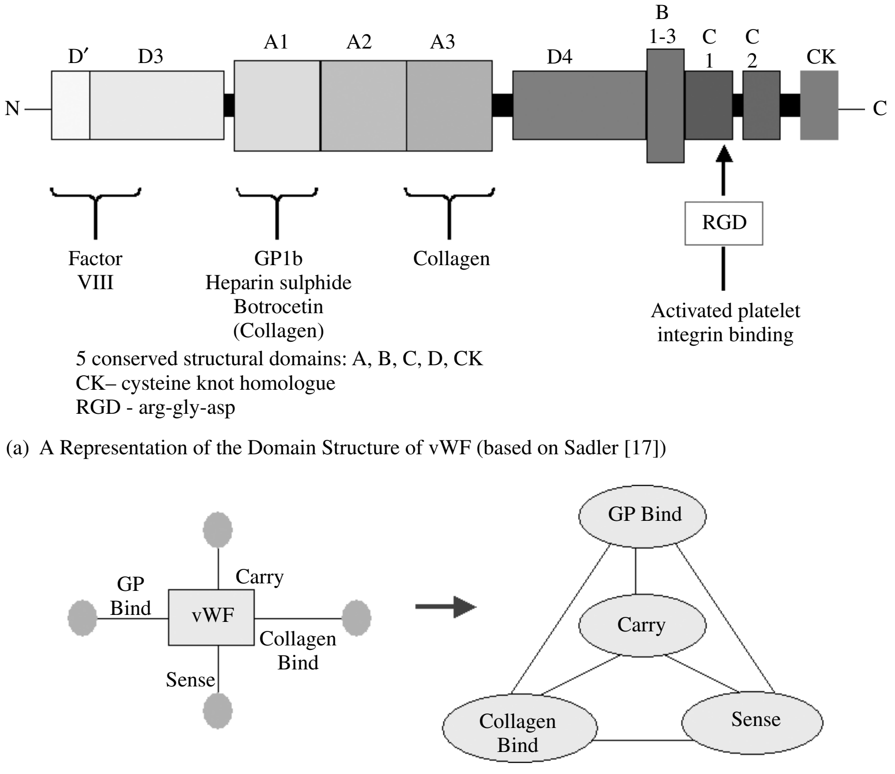

(a) A Representation of the Domain Structure of vWF (based on Sadler [17])

(b) Some Graphical Representations of vWF Functionality

**Figure 1.** VWF function

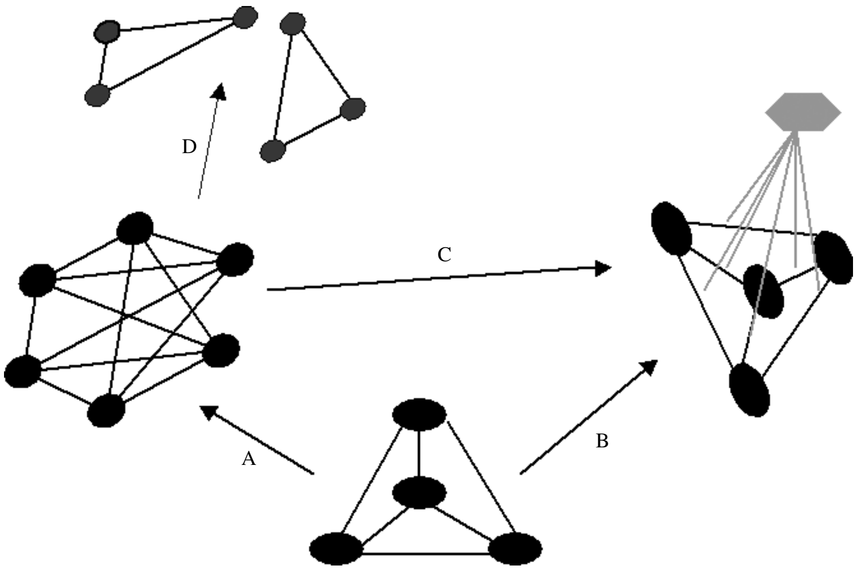

**Key**

A Transformation of Tetrahedron Graph to its Line Graph

B Visualisation of Pattern of Interactions between Tetrahedron Graph Processes

C Colimit of Transitive Patterns of Interaction Explicit in Line Graph

D Identification of Key Simplex Patterns in Line Graph

**Figure 2.** Some abstractions on graphs the source (tetrahedron) graph are related together. There are important conceptual relations between functionality and cohesion (implicit in the Karp and GO descriptions noted above). Ideas from the mathematical theory of categories can be used to illustrate this point, particularly pattern, co-limit and limit [2,4]. A pattern (diagram) is a collection of cooperating objects. The functional domains are interacting. The internal organization of a protein can by modelled by a pattern of domains (cooperating objects) in which links represent functional relations. A co-limit (cohesive binding) glues a pattern into a single unity in which the degrees of freedom of the parts are constrained by the whole. A limit represents the relationship between whole (i.e. the single unity) and its components.

Given the previous discussion, it is now possible to reason about functions with regard to how a whole is integrated or coheres out of its parts. Part–whole relations may be described as '*emergent cohesion*', reflecting an emergent or internal synergy in which interactions and/or local measurements generate cohesion. In category theoretic terms we use the idea of a co-limit, in terms of the descriptive dimensions of the concept of function (Table 1) we can use ideas like 'job', 'niche' and 'factor'. Cohesion concerned with whole–part relations is '*holistic cohesion*' in the sense that the whole keeps the parts together. This can be seen, for example, in welfare notions of functionality (Table 1).

### Concluding remarks

The function concept is complex and has many uses. We have considered certain aspects of this usage with regard to some exploratory tools that have been applied to a brief (re)description of vWF, viz. metaphors and verbs, the computational metaphor and diagrammatic graphs.

### References

1. Bray D. 1995. Protein molecules as computational elements in living cells. *Nature* **376**: 307–312.
2. Brown R, Paton RC, Porter TM (in press). Categorical language and hierarchical models for cell systems. In

Copyright © 2004 John Wiley & Sons, Ltd. 

*Computation in Cells and Tissues: Perspectives and Tools of Thought*, Paton R, Bolouri H, Holcombe M, Parish JH, Tateson R (eds). Series in Natural Computing. Springer: Heidelberg.
3. Conrad M. 1992. The seed germination model of enzyme catalysis. *BioSystems* **27**: 223–233.
4. Ehresmann AC, Vanbremeersch J-P. 1987. Hierarchical evolutive systems... *Bull Math Biol* **49**(1): 13–50.
5. Fisher M, Paton RC, Matsuno K. 1999. Intracellular signalling proteins as 'smart' agents in parallel distributed processes. *BioSystems* **50**: 159–171.
6. Gene Ontology Consortium. 2001. Creating the Gene Ontology resource: design and implementation. *Genome Res* **11**(8): 1425–1433.
7. Granit R. 1977. *The Purposive Brain*. MIT Press: Cambridge, MA.
8. Karp PD. 2000. An ontology for biological function based on molecular interactions. *Bioinformatics* **16**(3): 269–285.
9. Krohn K, Langer R, Rhodes J. 1967. Algebraic principles for the analysis of a biochemical system. *J Comp Syst Sci* **1**: 119–136.
10. Mayr E. 1982. *The Growth of Biological Thought*. Belknap/Harvard: Cambridge, MA.
11. Paton RC. 1997. Glue, verb and text metaphors in biology. *Acta Biotheor* **45**: 1–15.
12. Paton RC. 2001. Conceptual and Descriptive Displacements for the Mobilisation of Multiple Models in Biological Domains. Unclassified Report, Los Alamos National Laboratory LAUR-01-19.
13. Rosen R. 1967. Two-factor models, neural nets and biochemical automata. *J Theor Biol* **15**: 282–297.
14. Sadler JE. 1998. Biochemistry and genetics of von Willebrand factor. *Annu Rev Biochem* **67**: 395–424.
15. Tanford C, Reynolds J. 2001. *Nature's Robots: A History of Proteins*. Oxford: Oxford University Press.
16. Welch GR, Keleti T. 1987. Is cell metabolism controlled by a molecular democracy or a supramolecular socialism? *Trends Biochem Sci* **12**: 216–217.
17. Welch GR. 1987. The living cell as an ecosystem: hierarchical analogy and symmetry. *Trends Ecol Evol* **2**: 10, 305–309.

Copyright © 2004 John Wiley & Sons, Ltd. 
